# Television viewing time and all-cause mortality: interactions with BMI, physical activity, smoking, and dietary factors

**DOI:** 10.1186/s12966-022-01273-5

**Published:** 2022-03-19

**Authors:** Christopher T. V. Swain, Julie K. Bassett, Allison M. Hodge, David W. Dunstan, Neville Owen, Yi Yang, Harindra Jayasekara, James R. Hébert, Nitin Shivappa, Robert J. MacInnis, Roger L. Milne, Dallas R. English, Brigid M. Lynch

**Affiliations:** 1 Cancer Epidemiology Division, Cancer Council Victoria, Melbourne, Australia; 2 Department of Physiotherapy, The University of Melbourne, Melbourne, Australia; 3 Centre for Epidemiology and Biostatistics, Melbourne School of Population and Global Health, The University of Melbourne, Melbourne, Australia; 4 Physical Activity Laboratory, Baker Heart and Diabetes Institute, Melbourne, Australia; 5 Behaviour, Environment and Cognition Research Program, Mary MacKillop Institute for Health Research, Australian Catholic University, Melbourne, Australia; 6 Centre for Urban Transitions, Swinburne University, Melbourne, VIC Australia; 7 Behavioural Epidemiology Laboratory, Baker Heart and Diabetes Institute, Melbourne, VIC Australia; 8 Centre for Alcohol Policy Research, La Trobe University, Bundoora, Melbourne, Australia; 9 Cancer Prevention and Control Program &, Department of Epidemiology and Biostatistics, Arnold School of Public Health, University of South Carolina, Columbia, SC 29208 USA; 10 Department of Nutrition, Connecting Health Innovations LLC, Columbia, SC 29201 USA; 11 Precision Medicine, School of Clinical Sciences at Monash Health, Monash University, Clayton, VIC Australia

**Keywords:** Prospective study, Sedentary behavior, Survival analysis

## Abstract

**Background:**

Higher levels of time spent sitting (sedentary behavior) contribute to adverse health outcomes, including earlier death. This effect may be modified by other lifestyle factors. We examined the association of television viewing (TV), a common leisure-time sedentary behavior, with all-cause mortality, and whether this is modified by body mass index (BMI), physical activity, smoking, alcohol intake, soft drink consumption, or diet-associated inflammation.

**Methods:**

Using data from participants in the Melbourne Collaborative Cohort Study, flexible parametric survival models assessed the time-dependent association of self-reported TV time (three categories: < 2 h/day, 2–3 h/day, > 3 h/day) with all-cause mortality. Interaction terms were fitted to test whether there was effect modification of TV time by the other risk factors.

**Results:**

From 19,570 participants, 4,417 deaths were reported over a median follow up of 14.5 years. More TV time was associated with earlier mortality; however, this relationship diminished with increasing age. The hazard ratio (HR) and 95% confidence interval (95% CI) for > 3 h/day compared with < 2 h/day of TV time was 1.34 (1.16, 1.55) at 70 years, 1.14 (1.04, 1.23) at 80 years, and 0.95 (0.84, 1.06) at 90 years. The TV time/mortality relationship was more evident in participants who were physically inactive (compared with active; p for interaction < 0.01) or had a higher dietary inflammatory index score (compared with a lower score; p for interaction = 0.03). No interactions were detected between TV time and BMI, smoking, alcohol intake, nor soft-drink consumption (all p for interaction > 0.16).

**Conclusions:**

The relationship between TV time and all-cause mortality may change with age. It may also be more pronounced in those who are otherwise inactive or who have a pro-inflammatory diet.

**Supplementary Information:**

The online version contains supplementary material available at 10.1186/s12966-022-01273-5.

## Background

Sedentary behavior, defined as any waking behavior characterized by an energy expenditure ≤ 1.5 metabolic equivalents (METs) while in a sitting, or reclining posture [1], has been associated with several adverse health outcomes, including earlier mortality [2]. Support for this comes from a growing number of experimental and epidemiological studies, which demonstrate negative physiological effects and health outcomes following higher volumes of uninterrupted sitting time [2, 3]. These associations appear to be biologically plausible as well as dose dependent [2, 3]. This evidence has been used to inform recent public health guidelines, which encourage people to limit time spent sitting and to replace some sitting time with physical activity of any intensity [2].

The effects of sedentary behavior on health outcomes may not be the same for all people. For example, while recent studies support that higher levels of sedentary behavior are associated with premature mortality, the magnitude of these effects was lower for those who had high levels of physical activity compared with those who were less active [4–6]. These findings indicate that, for some, high levels of physical activity may attenuate the negative effects of sedentary behavior. The findings are also important as they suggest that for people who cannot, or will not, engage in health-improving behaviors such as moderate to vigorous physical activity, it is still possible to achieve a health benefit by reducing sedentary time. This allows for more informed, targeted, and resource-efficient health-promotion strategies.

There are reasonable grounds to suggest that physical activity may not be the only exposure that could modify the effects of sedentary behavior. Other modifiable exposures, such as body mass index (BMI), smoking, and diet also may interact with sedentary behavior to affect health. Some evidence for this has been identified in adolescents, where the association between higher screen time and cardiometabolic risk markers was less pronounced for those with a body mass index (BMI = weight(kg)/height(m)^2^) in the normal range (18.5 to < 25 kg/m2) compared with those who were overweight or obese (≥ 25 kg/m2) [7]. While sitting time has been found to be unrelated to cancer mortality in adult non-smokers, the magnitude of risk for cancer mortality in smokers progressively increased with higher sitting time [8]. This may be explained, in part, by an interaction between the pro-inflammatory effect of sedentary behavior and the carcinogens and other chemicals from smoking [8]. However, those findings were presented more as hypothesis generating, than definitive [8], and, collectively, the evidence for how sedentary behavior interacts with other modifiable exposures is limited [2]. Finally, in a large meta-analysis that examined associations between sedentary behaviors and all-cause mortality, effect estimates were stronger for TV time than for total sitting time, despite the similarity of the two exposures. The authors speculated that the difference may be attributable, in part, to snacking and TV food advertising that can influence diet [6]. There is also evidence for the potential of diet associated inflammation to modify the effect of other factors that work through inflammation-related pathways [9].

## Methods

### Aims

We aimed to assess the relationship between TV time and all-cause mortality and whether this is modified by BMI, physical activity, smoking, alcohol consumption, soft drink consumption, or the dietary inflammatory index.

### The Melbourne Collaborative Cohort Study

**Fig. 1:**
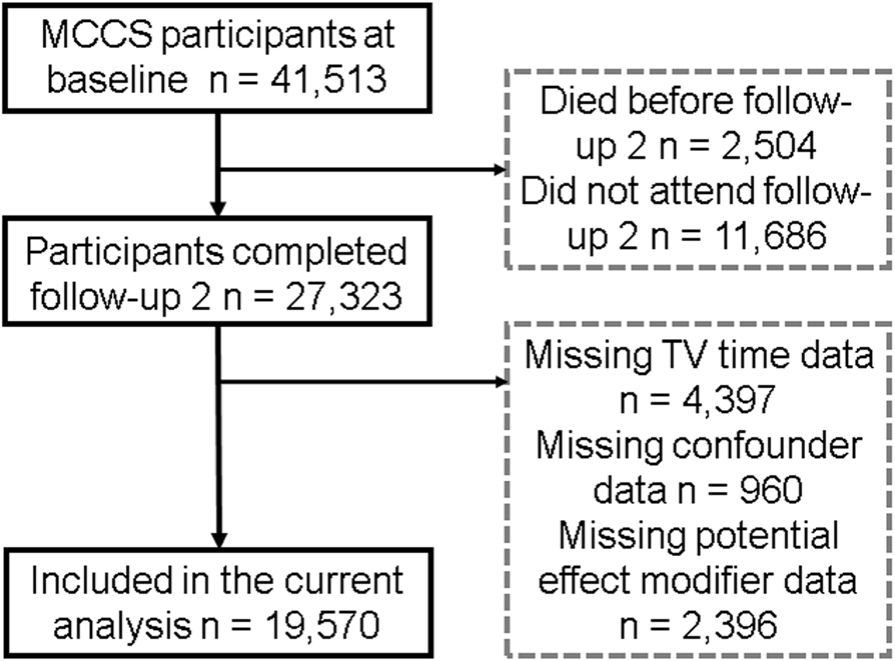
Participant selection

The Melbourne Collaborative Cohort Study (MCCS) is a prospective study that was undertaken to investigate relationships between socio-demographic factors, lifestyle patterns, diet, and the risk of developing cancer or other health outcomes [10, 11]. In brief, 41,513 (24,469 female, 17,044 male) adults predominantly aged between 40 and 69 years were recruited from the Melbourne metropolitan area in 1990 – 1994 (baseline). Southern European migrants were deliberately over-sampled to extend the range of dietary and lifestyle exposures [11]. For these analyses we used data from those who participated in the second wave of follow up (*n* = 27,323, 2003 – 2007), as this was the first occasion on which TV viewing time data were collected. Participants who were missing data for TV time, confounders, or potential effect modifiers were excluded from this analysis (Fig. 1). The study protocol was approved by Cancer Council Victoria’s Human Research Ethics Committee and all participants provided written informed consent [10, 11].

### TV time

TV time data were collected at the follow-up 2 visit (FUP2) by trained interviewers using a structured interview schedule. Participants reported the total time spent watching TV on week and weekend days. The average number of hours spent watching TV per day was calculated. Participants were categorized into three groups according to TV time: < 2 h/day, 2-3 h/day, and > 3 h/day.

### Potential effect modifiers

Data for potential effect modifiers were collected via physical measurement, interviewer administered questionnaire, or self-report questionnaire [11]. To calculate BMI (kg/m^2^), we used participant body mass (kg) measured at FUP2 with digital electronic scales, and height (m), recorded at baseline using a stadiometer.

Physical activity data were collected at FUP2 by interviewers using the Long Form International Physical Activity Questionnaire (IPAQ-Long). The total amount of physical activity performed for transport and leisure was calculated as per IPAQ-short guidelines and expressed in continuous form as weighted minutes per week [12]. Categorically, participants were classified as inactive (no activity), insufficiently active (< 150 min per week of activity), and sufficiently active (≥ 150 min/ week of activity) as per public health guidelines [13].

Participants who reported currently smoking at least seven cigarettes weekly at FUP2 were categorized as current smokers. Those not currently smoking but who had smoked at least seven cigarettes weekly for at least a year at baseline were categorized as ex-smokers. Others were classified as never smokers [14].

Alcohol intake at FUP2 was determined based on the frequency and quantity of intake per drinking occasion for beer, wine, and spirits during the previous year. Participants were categorized according to alcohol intake as non-drinkers (0 g/ day), light to moderate drinkers (> 0—< 40 g/ day), and heavy drinkers (≥ 40 g/ day) [15].

Dietary data, including soft drink intakes, were collected via self-report with a Food Frequency Questionnaire (FFQ) at FUP2 [16]. The energy-adjusted dietary inflammatory index score was calculated using responses for 29 foods and nutrients (out of a possible 45 items) for which intake values were available from the FFQ [17–19]. As a continuous measure, a negative score indicates a healthier, more anti-inflammatory diet whereas a more positive score indicates a less healthy, more pro-inflammatory diet. In a previous MCCS analysis, those who had a lower dietary inflammatory index typically consumed more olive oil, whole meal bread, fruit, and vegetables, as well as less red meat than those with a higher score [18]. As a categorical variable, we divided participants according to tertiles of dietary inflammatory index: ‘Lower’ (≤ -1.5), ‘Medium’ (-1.49 to -0.2), ‘Higher’ (> -0.2).

To measure soft drink consumption at FUP2, participants were asked how many glasses of regular (i.e., not diet) soft drink they consumed a day. Participants were divided into three categories based on glasses consumed per day: 0 glasses/ day, > 0 to < 1 glasses/ day, and ≥ 1 glass/ day.

### Mortality

Vital status was ascertained via data linkage to the Victorian Registry of Births, Deaths and Marriages and the National Death Index. Deaths until 31 December 2019 were included in the analysis, which was the date of the most recent registry data linkage at the time of analysis.

### Confounders

Selection of confounders was guided by a directed acyclic graph that was informed by existing literature (Supplement 1). Confounders included sex (male, female), the Socioeconomic Index for Areas for Disadvantage (SEIFA, quintiles), education (some/completed primary school, some high school, completed high school, tertiary education), marital status (married/de facto, single, divorced/separated, widowed), country of birth (grouped as Australia/ New Zealand, Northern Europe, Southern Europe), and history of angina, diabetes, heart attack, hypertension, or stroke (yes, no). SEIFA, a measure of socioeconomic status, was developed by the Australian Bureau of Statistics and ranks areas in Australia according to relative socio-economic advantage and disadvantage based on census information. To reflect temporal sequencing and to ensure these were confounders, we used baseline data for these measures. Potential effect modifiers, as described above, were also considered confounders.

### Statistical analyses

Descriptive statistics are presented as means and standard deviations (SD) for continuous variables and number (n) and percentage (%) for categorical variables.

Participants were followed from FUP2 to death or the date of the latest linkage (31 December 2019). In all models, age was used as the underlying time metric.

Flexible parametric survival models were fitted, using restricted cubic splines with three degrees of freedom (two knots placed at the 33^rd^ and 67^th^ percentiles) to model the baseline hazard for the relationship between TV time (categories) and all-cause mortality [20]. These are used to estimate hazard ratios (HR) and 95% confidence intervals (95% CI) as an estimate of the effect of an exposure on an outcome [20].

Models included sex, country of birth, education, marital status, SEIFA, CVD comorbidities, BMI, physical activity, smoking, alcohol consumption, the dietary inflammatory index, and soft drink consumption as outlined above. Visual inspection of log–log plots suggested that TV time, physical activity, and soft drink consumption had non-proportional hazards. Therefore, these were modelled as variables with time varying effects. Flexible parametric models allow for exposures and confounders to have time varying effects by fitting interactions between the variable and time using a second spline function that typically has fewer degrees of freedom. We modelled these using two degrees of freedom [20].

To assess effect modification of TV time, models were fitted with two-way interaction terms for TV viewing time and each potential effect modifier (i.e., physical activity, BMI, smoking, alcohol intake, soft drink consumption, and the dietary inflammatory index) and were compared with models that did not include the interaction term using the likelihood ratio test [21]. TV time and each potential effect modifier were modelled as categories. Models were adjusted for the same confounders as above and separate models were fit for each interaction term.

The ‘stpm2’ and ‘standsurv’ commands in Stata were used to generate time-varying hazard ratios for the main effects of TV time and all-cause mortality and for TV time and mortality by levels of significant effect modifiers [22, 23]. We report HR (95% CI) at three ages: 70, 80, and 90 years. For sensitivity analysis, we excluded participants with a BMI < 20 kg/m^2^ when testing for effect modification of BMI on TV time. This was done to remove potential bias for poor health associated with a low BMI. All statistical analyses were performed using Stata version 16 (Stata Corporation, College Station, Texas, USA).

## Results

Participant selection is presented in Fig. 1. There were 19,570 participants with complete TV time, confounder, and potential effect modifier data. TV time was not collected in earlier iterations of FUP2 (initial data collection focused on total sitting), which explains the large number of missing exposure data. Compared with those who did not participate in FUP2 or who were excluded due to missing FUP2 data, participants included in the analyses were more likely to be born in Australia, to have completed tertiary education, live in a less disadvantaged area at baseline, and less likely to have cardiometabolic conditions at baseline (Supplement 2). From the total of 19,570 participants, there were 4,517 deaths reported over a median follow up time of 14.5 years.

Participant descriptive data are presented in Table 1. The mean (SD) age of the sample at FUP2 was 66 (9) years. Compared with participants who watched the most TV, those who watched the least TV were, on average, slightly younger, more likely to have a tertiary qualification and to live in a less-disadvantaged area, and less likely to have a history of cardiometabolic conditions. There were 618 (3%) participants who were underweight and excluded in sensitivity analyses.

**Table 1:** Participant characteristics. Data are presented as mean (SD) for continuous variables and frequency (%) for categorical variables

	By TV time category			Entire sample
	< 2 h/ week	2–3 h/ week	> 3 h/ week	
Total *n (%)*	5,738 (29)	8,202 (42)	5,630 (29)	19,570
Age at FUP2 (*y), mean (SD)*	63 (8.6)	66 (8.6)	68 (8.4)	66 (8.7)
BMI (kg/m^2^), *mean (SD)*	27 (4.5)	27 (4.6)	28 (5.0)	27 (4.7)
TV time (hrs/week), *mean (SD)*	1.3 (0.49)	2.5 (0.42)	4.4 (1.2)	2.6 (1.5)
Female, *n (%)*	3,334 (58)	5,033 (61)	3,480 (62)	11,847 (61)
Country of Birth				
Australia/ New Zealand, *n (%)*	4,304 (75)	6,223 (76)	4,412 (78)	14,939 (76)
Northern Europe *n (%)*	397 (7)	633 (8)	363 (7)	1,393 (7)
Southern Europe *n (%)*	1,037 (18)	1,346 (16)	855 (15)	3,238 (17)
Education				
Some/ completed primary school*, n (%)*	652 (11)	905 (11)	688 (13)	2,245 (11)
Some high school/ technical school*, n (%)*	1,544 (27)	3,240 (40)	2,837 (51)	7,621 (39)
Completed high school/ technical school*, n (%)*	547 (10)	924 (11)	600 (11)	2,071 (11)
Tertiary/ diploma/ degree*, n (%)*	2,995 (52)	3,133 (38)	1,505 (27)	7,633 (39)
Socioeconomic Index for Areas of Disadvantage				
1^st^ Quintile (higher disadvantage)*, n (%)*	699 (12)	1,079 (13)	986 (18)	2,764 (14)
2^nd^ Quintile*, n (%)*	879 (15)	1,347 (16)	1,080 (19)	3,306 (17)
3^rd^ Quintile*, n (%)*	813 (14)	1,220 (15)	868 (15)	2,901 (15)
4^th^ Quintile*, n (%)*	1,194 (21)	1,712 (21)	1,158 (21)	4,064 (21)
5^th^ Quintile*, n (%)*	2,153 (38)	2,844 (35)	1,538 (27)	6,535 (33)
Marital Status				
Married/ De Facto*, n (%)*	4,363 (76)	6,295 (77)	4,131 (73)	14,789 (76)
Single*, n (%)*	545 (10)	644 (8)	446 (8)	1,635 (8)
Divorced/ Separated*, n (%)*	611 (11)	751 (9)	573 (10)	1,935 (10)
Widowed*, n (%)*	219 (4)	512 (6)	480 (9)	1,211 (6)
Cardiometabolic Comorbidities				
Yes, *n (%)*	1,011 (18)	1,828 (22)	1,559 (28)	4,398 (23)
Physical activity				
Inactive*, n (%)*	305 (5)	517 (6)	526 (9)	1,348 (7)
Insufficiently active*, n (%)*	1,183 (21)	1,796 (22)	1,423 (25)	4,402 (23)
Sufficiently active*, n (%)*	4,250 (74)	5,889 (72)	3,681 (65)	13,820 (71)
Smoking status				
Never, *n (%)*	3,678 (64)	5,102 (63)	3,391 (60)	12,171 (62)
Former, *n (%)*	1,807 (32)	2,705 (33)	1,887 (34)	6,399 (33)
Current, *n (%)*	257 (5)	377 (5)	352 (6)	986 (5)
Alcohol intake				
Non-drinker, *n (%)*	1,590 (28)	2,516 (31)	2,060 (37)	6,166 (32)
Light/ moderate drinker, *n (%)*	3,813 (67)	5,201 (63)	3,239 (58)	12,253 (63)
Heavy drinker, *n (%)*	335 (6)	485 (6)	331 (6)	1,151 (6)
Energy-Adjusted Dietary Inflammatory Index				
Lower (≤ -1.5), *n (%)*	2,149 (38)	2,738 (33)	1,611 (29)	6,498 (33)
Medium (-1.49 to -0.2), *n (%)*	1,875 (33)	2,758 (34)	1,891 (34)	6,524 (33)
Higher (≥ -0.2), *n (%)*	1,714 (30)	2,706 (33)	2,128 (38)	6,548 (34)
Soft drink consumption				
No consumption, *n (%)*	3,266 (57)	4,493 (55)	2,998 (53)	10,757 (55)
< 1 glass/ day, *n (%)*	2,098 (35)	3,014 (37)	2,052 (37)	7,164 (37)
≥ 1 glass/ day, *n (%)*	380 (7)	695 (9)	580 (10)	1,655 (8)

**Fig. 2:**
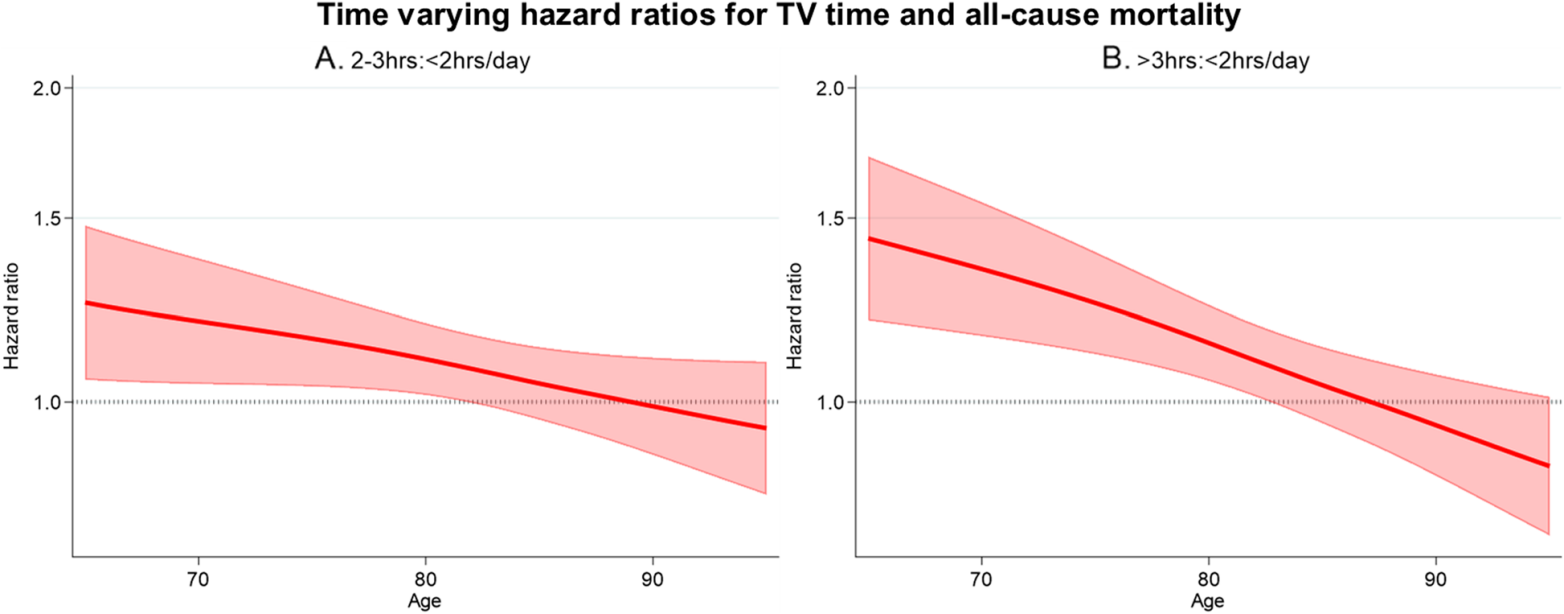
Time varying HR for **A**. 2-3 h/day; and **B**. > 3 h/day of TV time and all-cause mortality. < 2 h/day was the reference category. The thick red line represents the HR, the shaded area represents the 95% CI. Age was the underlying time metric. Models included alcohol consumption, BMI, country of birth, CVD comorbidities, the dietary inflammatory index, education, marital status, physical activity, SEIFA, sex, smoking, and soft drink consumption

The time-varying HRs (95%CI) for TV time and all-cause mortality are presented in Fig. 2. Higher TV time was associated with increased all-cause mortality; however, this effect decreased with increasing age. The HR (95% CI) for 2–3 h of TV compared with < 2 h of TV was 1.19 (1.04, 1.37) at 70 years, 1.10 (1.01, 1.19) at 80 years, and 0.99 (0.89, 1.06) at 90 years. For > 3 h compared with < 2 h of TV time, the HR (95%CI) was 1.34 (1.16, 1.55) at 70 years, 1.14 (1.04, 1.23) at 80 years, and 0.95 (0.84, 1.06) at 90 years.

Significant interactions between TV time and physical activity (*p* =  < 0.01) as well as the dietary inflammatory index (*p* = 0.03) were identified. There were no interactions identified between TV time and BMI (*p* = 0.16), smoking (*p* = 0.82), alcohol intake (*p* = 0.29), or soft drink consumption (*p* = 0.56). Results did not appreciably change after excluding participants who had a BMI < 20 kg/m^2^ (not shown).

**Fig. 3:**
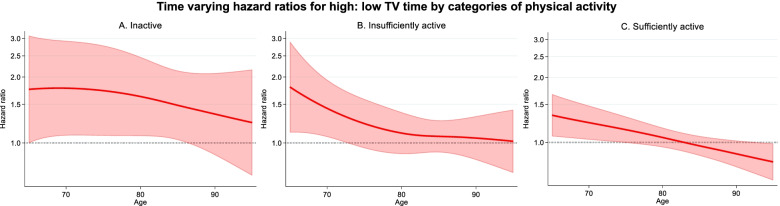
Time varying HR for > 3 h/ day TV time and all-cause mortality when participants were **A** Physically inactive; **B** Insufficiently active; and **C** Sufficiently active. < 2 h/day TV time was the reference category. The thick red line represents the HR, the shaded area represents the 95% CI. Age was the underlying time metric. Adjusted for alcohol consumption, BMI, country of birth, CVD comorbidities, education, the dietary inflammatory index, marital status, SEIFA, sex, smoking, and soft drink consumption

The time varying HR (95%CI) for TV time and all-cause mortality by categories of physical activity are presented in Fig. 3. Higher levels of TV time were associated with increased risk of mortality for participants who were either inactive or insufficiently active. For inactive participants, the HR (95% CI) for > 3 h/ day compared with 2 h/ day was 1.78 (1.08, 2.92) at 70 years, 1.63 (1.08, 2.45) at 80 years, and 1.35 (0.88, 2.08) at 90 years. For insufficiently physical activity, these HR (95% CI) were 1.43 (1.06, 1.92) at 70 years, 1.11 (0.89, 1.37) at 80 years, and 1.05 (0.83, 1.33) at 90 years. TV time – mortality HRs were reduced for those who were sufficiently active. The HR (95% CI) for > 3 h/ day compared with < 2 h/ day of TV time was 1.23 (1.03, 1.47) at 70 years, 1.05 (0.95, 1.17) at 80, and 0.90 (0.79, 1.03) at 90 years.

**Fig. 4:**
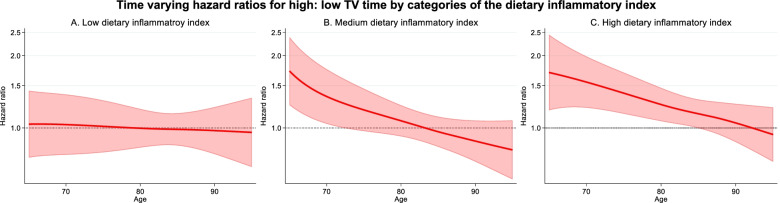
Time varying HR for > 3 h/ day TV time and all-cause mortality when participants had a **A** Low energy adjusted dietary inflammatory index, **B** Medium dietary inflammatory index, and **C** High dietary inflammatory index. < 2 h/day TV time was the reference category. The thick red line represents the HR, the shaded area represents the 95% CI. Age was the underlying time metric. Adjusted for alcohol consumption, BMI, country of birth, CVD comorbidities, education, marital status, physical activity, SEIFA, sex, smoking, and soft drink consumption

The time varying HR (95%CI) for TV time and all-cause mortality by categories of the dietary inflammatory index are presented in Fig. 4. TV time was not associated with mortality in those with a low (< -1.5) dietary inflammatory index. The HR (95% CI) for > 3 h/ day compared with 2 h/ day was 1.03 (0.71, 1.38) at 70 years, 1.00 (0.83, 1.20) at 80 years, and 0.97 (0.78, 1.22) at 90 years. For a medium dietary inflammatory index (-1.49 to -0.2) these HR (95% CI) were 1.35 (1.04, 1.75) at 70 years, 1.07 (0.92, 1.24) at 80 years, and 0.88 (0.72, 1.07) at 90 years. For a higher dietary inflammatory index (> -0.2), more TV time was associated with higher mortality. The HR (95% CI) for > 3 h of TV time compared with 2 h in participants with a high dietary inflammatory index was 1.55 (1.21, 1.98) at 70 years, 1.25 (1.07, 1.46) at 80 years, and 1.05 (0.87, 1.26) at 90 years.

## Discussion

Compared with less TV time, more time spent watching TV was associated with earlier mortality. This relationship was age dependent as the HR for TV time diminished with age. The negative effects of TV time were most evident in people who were physically inactive or who had a more pro-inflammatory diet. No effect modification by BMI, smoking, alcohol intake, or soft drink consumption was identified.

Strengths of our study include a large cohort of adults with data collected on a wide range of lifestyle factors, 14 years of follow up, and use of flexible parametric models to examine the time varying relationship between TV time and mortality. However, there are several limitations that should be considered when interpreting our findings. That we could only examine TV time at FUP2 does introduce selection bias, i.e., participants who were more vulnerable to the ill-effects of sedentary behavior may not have participated in FUP2. MCCS participants with TV time data were more likely to live in less disadvantaged areas, have a higher level of education, and were less likely to have cardiometabolic comorbidities at baseline compared with participants who did not participate in FUP2 or provide TV time data. In addition, although assessment of TV time has been shown to be reliable, there may be measurement error in this self-reported data for TV time or potential effect modifiers that could attenuate the findings [24]. Further, as TV time was measured once only, we cannot eliminate the possibility of exposure departures over the 14-year follow up period. The potential for reverse causation must also be acknowledged, as those who are unwell may be more likely to watch more TV and engage in less physical activity. We looked to address this by adjusting for pre-existing cardiometabolic diseases.

An association between more TV time and earlier mortality is consistent with prior research. Meta-analyses have identified increased risk of all-cause, cardiovascular, and cancer mortality with increased sedentary behavior and specifically, time spent watching TV [4, 25]. We have additionally shown that the HR for TV time and all-cause mortality decreased by increasing age. This may indicate that participants who watched more TV were less well and more likely to die sooner, that behavior changed over time and the negative effects of sedentary behavior may be reversible, that those who live longer are those who are more robust to the impacts of high TV time on its own, or that TV time is not relevant in terms of mortality at age 90.

The relationship between TV time and mortality was most evident in those who were physically inactive but was much less evident in those who were sufficiently active. This suggests that sedentary behavior reduction interventions may be most effective when they target those who are otherwise physically inactive. The finding is consistent with prior research, which has identified that physical activity can attenuate some of the deleterious effects of sedentary behavior [5, 6]. A prior harmonized meta-analysis with data from more than one-million participants [6], reported that higher levels of physical activity reduced but did not eliminate the association between long TV time and earlier mortality. These findings emphasize that reducing sedentary behavior in more physically active people should not be neglected.

The relationship between TV time and mortality was more evident in participants who consumed a more pro-inflammatory rather than an anti-inflammatory diet. This suggests that diets with anti-inflammatory properties may counter the negative effects of higher TV viewing time. This finding is of note, as inflammation is one of the underlying mechanisms proposed to link sedentary behavior with adverse health outcomes, including mortality [3, 8], with regular muscle contraction considered necessary for optimal regulation of inflammation [26]. To our knowledge, this is the first study that has identified effect modification of TV time by the dietary inflammation index. It is also possible that remaining sedentary by watching TV in the evening after dinner may negatively affect glucose and lipid metabolism [27], and this could be especially problematic for diets of lower quality. The interaction between TV time and the dietary inflammatory index also highlights the importance of targeted health interventions. These may include reducing unhealthy snacking that is common during watching TV and looking to mitigate the negative effects of TV advertising campaigns on diet quality [28, 29].

The absence of interactions between TV time and BMI, smoking, alcohol, or soft drink consumption was unexpected. Experimental studies have demonstrated that the negative metabolic and endothelial effects of sugary drinks are greater after sedentary lying compared with exercise [30]. Observational studies have identified modification of the screen time – cardiometabolic risk factor relationship by BMI and increased risk of cancer mortality with more time spent watching TV in smokers [7, 8]. To some extent, limitations in the data should be considered here. For instance, more than half of our participants reported no soft-drink consumption and this behavior is generally more common in younger age groups [31]. In addition, the dose-dependent relationship between alcohol intake and mortality is complex and non-linear [32], and this may have prevented identification of effect modification by categories of alcohol consumption. However, when considering that the association between TV time alone and mortality was small and time dependent, a reasonable interpretation may be that the relatively large effects of obesity, smoking, or alcohol intake overwhelm those of TV time.

Further studies are needed to determine whether our findings, and the absence of some significant findings, for the interactions between TV time and BMI, smoking, and dietary factors are replicable. Cohort studies with multiple assessments of TV time may provide further insight into the time-varying relationship between TV time and health outcomes such as mortality. Exploring these interactions among different populations or different forms of sedentary behavior may provide additional insight into who and what combination of behaviors health interventions should target. As, in our study, those who watched the most TV generally attained lower levels of education and lived in areas of greater disadvantage, we suggest focus on these groups should be prioritized.

## Conclusion

In this large prospective cohort study of metropolitan-Australian adults, more TV time was associated with earlier all-cause mortality; however, this relationship diminished with age. A relationship between TV time and mortality was most evident in participants who were inactive or who had a high dietary inflammatory index. An absence of interactions between TV time and BMI, smoking, or alcohol consumption may imply that the risks derived from these behaviors is so high that the influence of TV time is rendered negligible.

## Supplementary Information

**Additional file 1.**

## Data Availability

Details on how to access data for the Melbourne Collaborative Cohort Study are available at: https://www.cancervic.org.au/research/epidemiology/pedigree

## Abbreviations

95% CI: 95% Confidence interval
BMI: Body mass index
FUP2: 2^Nd^ study follow up
HR: Hazard ratio
MCCS: Melbourne Collaborative Cohort Study
IPAQ: International physical activity questionnaire
SIEFA: Socioeconomic Index for Areas of Disadvantage
TV time: Television viewing time
