# Supplementary material for: Television viewing time and all-cause mortality: interactions with BMI, physical activity, smoking, and dietary factors
Source: Int J Behav Nutr Phys Act. 2022 Mar 19;19:30. doi: 10.1186/s12966-022-01273-5 (PMC8934515; doi:10.1186/s12966-022-01273-5)
Supplement: Supplementary file 1 — Additional file 1. [file 12966_2022_1273_MOESM1_ESM.docx]

Television viewing time and all-cause mortality: Supplementary material

**
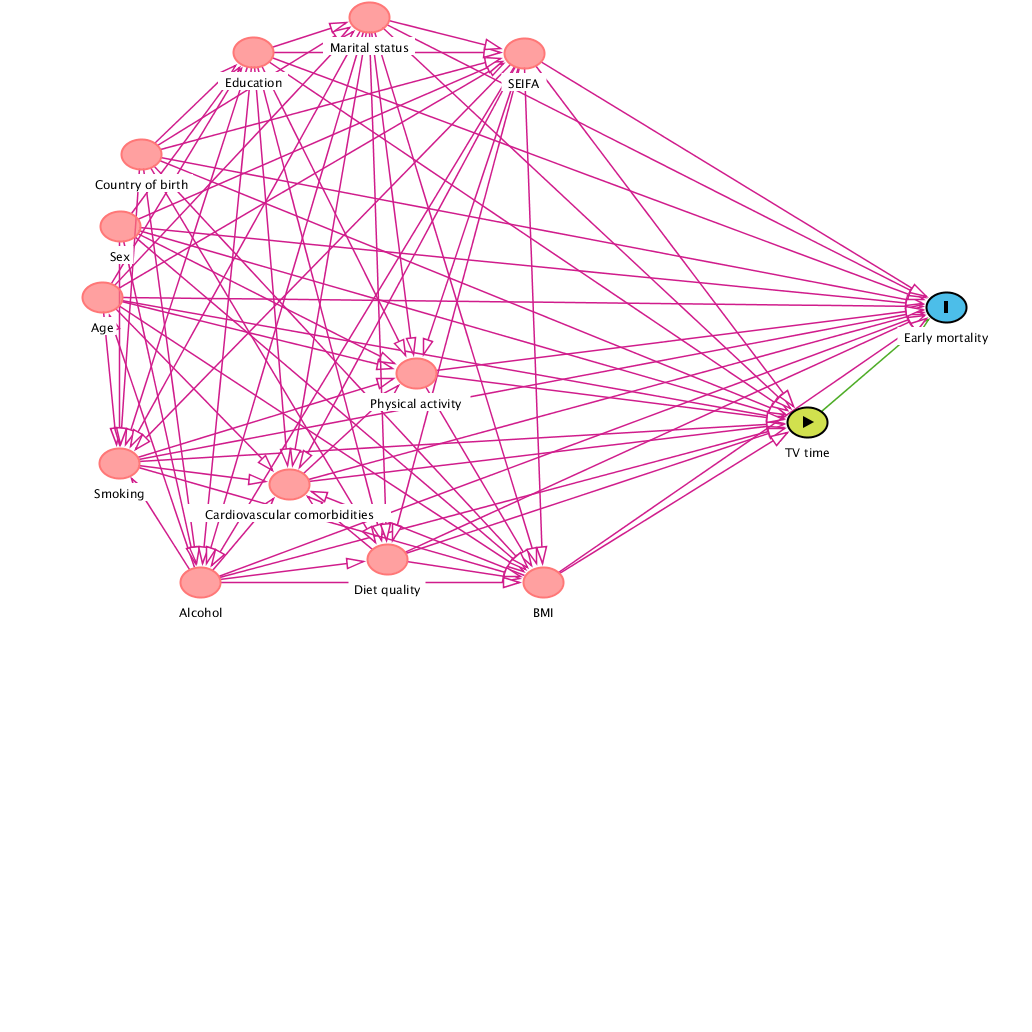
**

**Supplementary figure 1:** Directed acyclic graph for the main effects of TV time on all-cause mortality

**Supplementary table 1:** Comparison of baseline characteristics for included and excluded MCCS participants

|  | Excluded | Included |
| --- | --- | --- |
| Total *n* | 21,943 | 19,570 |
| Baseline age, *y (sd)* | 56.5 (8.7) | 54 (8.5) |
| Female, *n (%)* | 12,622 (57) | 11,847 (61) |
| Country of Birth |  |  |
| Australia/ New Zealand, *n (%)* | 13,575 (62) | 14,939 (76) |
| Northern Europe *n (%)* | 1,256 (6) | 1,393 (7) |
| Southern Europe *n (%)* | 7,112 (32) | 3,238 (17) |
| Education |  |  |
| Primary school*, n (%)* | 5,801 (27) | 2,245 (12) |
| Some high school/ technical school*, n (%)* | 8,232 (38) | 7,621 (40) |
| Completed high school/ technical school*, n (%)* | 2,052 (9) | 2,071 (11) |
| Tertiary/ diploma/ degree*, n (%)* | 5,899 (27) | 7,633 (39) |
| Socioeconomic Index for Areas of Disadvantage |  |  |
| 1^st^ Quintile*, n (%)* | 4,849 (22) | 2,764 (14) |
| 2^nd^ Quintile*, n (%)* | 5,415 (25) | 3,306 (17) |
| 3^rd^ Quintile*, n (%)* | 3,618 (17) | 2,901 (15) |
| 4^th^ Quintile*, n (%)* | 3,509 (16) | 4,064 (21) |
| 5^th^ Quintile*, n (%)* | 4,301 (20) | 6,535 (33) |
| Marital Status |  |  |
| Married/ De Facto*, n (%)* | 14,457 (72) | 14,789 (76) |
| Single*, n (%)* | 1,980 (10) | 1,635 (8) |
| Divorced/ Separated*, n (%)* | 2,093 (10) | 1,935 (10) |
| Widowed*, n (%)* | 1,590 (8) | 1,211 (6) |
| Cardiometabolic Comorbidities |  |  |
| Yes, *n (%)* | 6,768 (31) | 4,398 (23) |
